# Comparing factors influencing wellbeing in young adults with aphasia and young adults with developmental language disorder

**DOI:** 10.1111/1460-6984.70020

**Published:** 2025-03-06

**Authors:** Vasiliki Kladouchou, Nicola Botting, Katerina Hilari

**Affiliations:** ^1^ Centre for Language and Communication Science Research City St George's, University of London London UK

**Keywords:** aphasia, developmental language disorder, influencing factors, wellbeing, young adults

## Abstract

**Background:**

Understanding factors influencing wellbeing is crucial for the development of effective services. Aphasia in older individuals and developmental language disorder (DLD) in children significantly affect how people live and function. Despite the increasing stroke incidence in young adults and the growing recognition of DLD as a lifelong disorder, the literature lacks evidence on the wellbeing of young adults, aged between 18 and 40 years old, with these conditions.

**Aims:**

To identify factors influencing wellbeing in young adults with aphasia and DLD.

**Methods & Procedures:**

This cross‐sectional between‐group study involved 78 young adults with aphasia, DLD and no language impairments, with mean (SD) age of 30.5 (6.38) years. A total of 12 measures were used to assess wellbeing, language, cognition, health, emotional distress, social functioning and psychological resources. Feasibility and acceptability were informed by a pilot‐study. One‐way‐unrelated analysis of variance (ANOVA) and Kruskal–Wallis tests were used to compare groups on language, cognition and wellbeing, while correlation analyses identified factors influencing wellbeing in each group.

**Outcomes & Results:**

No significant differences were found between clinical groups in language and cognitive profiles, with both scoring lower than those with no language impairments. The wellbeing of individuals with aphasia and DLD was similar to that of adults with no language impairments. Health, emotional distress and social support were common drivers of wellbeing among all groups, with positive performance in those scales indicating better wellbeing. Language and self‐esteem showed significant links with wellbeing for those with DLD, but not for the aphasia group. The higher the self‐esteem level of people with DLD, the higher their wellbeing rating. Interestingly, language was negatively related to wellbeing in people with DLD, with higher language scores correlating with lower levels of wellbeing.

**Conclusions & Implications:**

Aphasia and DLD share factors affecting wellbeing, but the different origins of the disorders seem to influence the overall nature of wellbeing. Wellbeing in DLD is primarily impacted by the language disorder, whereas in aphasia it is influenced by its secondary to the disorder characteristics and primarily emotional health. This study highlights the need for holistic therapy and ongoing psychosocial support to optimize services for young adults with these impairments.

**WHAT THIS STUDY ADDS:**

**What is already known on the subject**

- Aphasia and DLD significantly impact social and emotional aspects of life. However, knowledge about wellbeing in young adults with these disorders remains limited.

**What this paper adds to the existing knowledge**

- This research is the first to compare wellbeing in aphasia and DLD. It highlights the impact of several psychosocial factors and clinical characteristics including language on wellbeing, considering whether individuals developed language disorders during childhood or acquired them later in adult life. While there is a small amount of evidence on wellbeing in young adults with DLD, this is the first study to investigate wellbeing in young adults with aphasia.

**What are the potential or actual clinical implications of this work?**

- The type of language disorder may influence wellbeing in diverse ways. Speech and language therapists must evaluate the impact of these disorders on young adults’ wellbeing. Rehabilitation should consider both personal factors and external support systems, following appropriate care models. Our findings suggest that aphasia interventions might benefit from focusing on enhancing emotional wellbeing and social networks, particularly in young adults. For individuals with DLD, it appears that the emphasis should be on improving communication skills by addressing functional constraints in social interactions and considering their psychological resources. Further research is needed to confirm these potential intervention strategies. An interdisciplinary approach is essential for optimal outcomes.

## INTRODUCTION

### Background

Understanding factors influencing wellbeing is crucial for designing effective services (Office for National Statistics (ONS), 2015). Language disorders, whether developmental or acquired, significantly impact affected individuals. Evidence shows that children and adolescents with developmental language disorder (DLD) and older individuals with aphasia encounter challenges in various aspects of life, including social roles and emotional health (Durkin & Conti‐Ramsden, 2010; Kauhanen et al., 2000; Northcott et al., 2016). Information on the specific effects of these challenges on overall wellbeing is essential for effectively addressing the needs of these clinical groups.

Early adulthood is crucial for wellbeing due to evolving social, career and family aspects (Schaapsmeerders et al., 2013). Still, information on young adults^1^ with aphasia and DLD is scarce, which is concerning given the rising stroke incidence in younger people (Béjot et al., 2014) and the recognition of DLD as a lifelong disorder with a long‐term impact (Conti‐Ramsden et al., 2018; Clegg et al., 2005).

Wellbeing is conceptualized in this paper as the ways in which people think, feel and function, both personally and socially, and how they evaluate their lives as a whole.

We developed and used the working definition of wellbeing above for this study, based on several well‐established wellbeing frameworks (Thompson & Marks, 2008; Whitehall Wellbeing Working Group, 2006, as cited in Steuer & Marks, 2008); Beaumont et al., 2012; Michaelson et al., 2012). This was necessary because, although research on wellbeing in the general population has expanded in recent years, there is no universal definition of the concept. Instead, researchers have primarily focused on its various dimensions rather than the concept itself (Dodge et al., 2012).

Wellbeing is particularly relevant for young adults with DLD and post‐stroke aphasia due to the specific challenges these groups face. Cognitive impairments and emotional difficulties are common in both populations, which can affect their ability to think and feel positively about themselves and their lives (Arkkila et al., 2008; Botting, Durkin et al., 2016; Clegg et al., 2005; Hilari et al., 2012). Additionally, communication barriers significantly impact their daily functioning, leading to difficulties in personal and social participation. These barriers can contribute to isolation and reduced opportunities for meaningful social interactions, further influencing their overall wellbeing (Lawrence, 2010; Toseeb et al., 2017).

Wellbeing and quality of life (QoL) are often used interchangeably and loosely defined in the literature, leading to considerable confusion (Diener & Suh, 1997; Muldoon et al., 1998; Frey & Stutzer, 2002). Many emotional and social concepts considered related to wellbeing in our project also fall under the umbrella term QoL (Shin & Johnson, 1978; Diener et al., 1999). However, often subtle distinctions are made between the two constructs. Subjective wellbeing, for instance, appears to encompass constructs such as life satisfaction, positive affect and negative affect, and is commonly used to assess an individual's appraisal of their health status and overall life circumstances (Gill, 1994; Rees et al., 2010). It emphasizes dimensions such as happiness, satisfaction and emotional wellbeing, and it aligns closely with the subjective experiences of individuals (Cummins, 2010).

On the other hand, the term QoL often encompasses various life aspects, including physical health, material wellbeing and life satisfaction, incorporating both objective and subjective evaluations (Felce & Perry, 1995). According to Dodge et al. (2012), a narrow emphasis on QoL may not adequately help to define wellbeing, as wellbeing is a broader concept. Wellbeing theories highlight the importance of individuals’ own perceptions and judgments of their QoL, underlining the subjective nature of wellbeing assessments (Stratham & Chase, 2010).

In this study we chose to investigate wellbeing due to its comprehensive nature. Wellbeing provides a nuanced understanding of the psychological and social functioning of individuals with communication disorders, capturing aspects particularly relevant for our client groups who often present with challenges in those areas (Botting, Toseeb et al., 2016; Hilari et al., 2012; Toseeb et al., 2017). Given the subjective nature of wellbeing, it allows us to explore unique experiences and perspectives of individuals with communication disorders, guiding targeted therapeutic interventions and support strategies (Keyes, 2002).

#### Post‐stroke aphasia and wellbeing

Strokes rank as the third leading cause of death in the UK (Wolfe, 2000), while their incidence has been rising in those under 55 over recent decades (Béjot et al., 2014). Specifically, 10–14% of ischemic strokes occur between ages 18 and 50 (Schaapsmeerders et al., 2013) driving greater interest in the outcomes for this age group (Nedeltchev et al., 2005).

Aphasia affects approximately 30% of stroke survivors (Flowers et al., 2013), significantly impacting their daily lives and overall QoL (Hilari, 2011). Although younger individuals have been included in studies, research has often been skewed towards older populations which is the typical demographic affected by stroke. For younger adults, aphasia has a profound influence on identity, relationships and social participation (Lawrence, 2010). However, when examining younger stroke survivors, the emphasis has often been on their return to work, neglecting other crucial aspects of their lives (Daniel et al., 2009).

Notably, the majority of stroke outcome research tends to concentrate on individuals who have had a stroke. It often does not specifically address those who experience aphasia (Patel et al., 2007). Given the unique challenges presented by aphasia, it remains uncertain to what extent findings from stroke studies without specific aphasia reporting can be applied to the aphasia population.

There is even more limited research on the determinants of wellbeing of young stroke patients. After conducting a systematic review on factors affecting wellbeing and related concepts in young adults with aphasia, we found only one study meeting our inclusion criteria. That study (Kim et al., 2005) found that factors such as disability, motor dysfunction and economic status impact QoL, with aphasia also correlating with decreased QoL (Kim et al., 2005). Even so, this study included only a small sample with aphasia and focused on QoL rather than wellbeing. There are studies that have focused on factors affecting QoL of young people with stroke. A recent scoping review on the topic included nine studies (Gurková et al., 2023). It found that QoL in young stroke patients was primary affected by functional status, independence, fatigue and depression. However, those do not report on people with aphasia, while the mean age of the participants is higher than 40 years old.

#### DLD and wellbeing

DLD is a lifelong condition affecting 7.6% of children (Norbury et al., 2016) and characterized by language impairments with no apparent cause (Bishop and Snowling, 2004).

Research indicates that young adults with DLD face increased emotional challenges, including depression and anxiety, and limited social interactions (Botting, Toseeb et al., 2016; Toseeb et al., 2017). While the origins of these challenges remain unclear, it is noteworthy that young adults with DLD align with their non‐DLD peers in terms of overall wellbeing (Conti‐Ramsden et al., 2016; Johnson et al., 2010; Records et al., 1992).

Although this may seem contradictory, it is important to note that emotional challenges, such as anxiety and depression, are recognized as central factors influencing wellbeing (Thompson & Marks, 2008) but are not synonymous with it. Wellbeing encompasses broader evaluations of how individuals think, feel and function in their lives. Research suggests that individuals can report good overall wellbeing even while experiencing emotional challenges, and vice versa.

Despite the adverse impact of language impairments on critical psychosocial life domains, the overall wellbeing of young adults with DLD appears comparable to that of their non‐DLD peers, This highlights a complex interplay where the negative effects of DLD may be mitigated by other factors, enabling these individuals to maintain their wellbeing. Identifying and understanding these mitigating factors could offer valuable insights into how young adults with DLD navigate their challenges and sustain a positive QoL.

In terms of predictors of wellbeing in young adults with DLD, one study explored this investigating three factors including health, employment and relationships and found that self‐rated health emerged as the most consistent predictor of personal wellbeing in relation to life satisfaction (21% of variance), happiness (11%) and perception of life's worthwhileness (32%) (Conti‐Ramsden et al., 2016). Other studies have examined various aspects of young adults’ lives, including emotional health (Botting, Toseeb et al., 2016), social functioning (Toseeb et al., 2017), and psychological factors such as self‐esteem and self‐efficacy (Durkin et al., 2017).

### Comparing wellbeing in aphasia and DLD

In our knowledge, the two populations under study have never been directly compared in research. Undertaking a comparative analysis of the factors influencing wellbeing in young adults facilitates the development of theoretical models about wellbeing in general, as well as informing support for these particular groups. A comparison of the two groups allows for a more comprehensive understanding of how these factors influence wellbeing across different types of communication disorders. By analysing these factors together, we can identify both commonalities and differences in the experiences of individuals with DLD and aphasia, allowing us to explore potential interactions between the conditions themselves and the influencing factors, which might not be apparent if the groups were analysed separately. Ultimately, this combined analysis provides a richer, more holistic view of how various factors contribute to wellbeing in individuals with communication disorders, guiding the development of more effective support strategies.

### Rationale and research questions

As noted above, we currently have some evidence regarding language profiles and psychosocial functioning in young adults with DLD and separately for older adults with aphasia. However, there are important unanswered questions: There is a lack of research measuring wellbeing per se in these groups; the evidence does not focus on young adults; and these populations have not yet been systematically compared on their wellbeing or their language and cognitive status. Also, links between overall wellbeing and other life factors remain unclear. The present study aims to address this gap in literature by assessing wellbeing and psychosocial variables in typical young adults with DLD and aphasia. We also include comprehensive assessment of background language and cognition skills to explore how these relate to wellbeing. Specifically, we ask the following research questions:

- How do the language and cognitive skills of young adults with aphasia, DLD and typical individuals compare?
- How does the wellbeing of young adults with aphasia, DLD and typical individuals compare?
- What are the shared and different influencing factors of wellbeing between young adults with aphasia, DLD and typical individuals?

## METHODS

### Ethics

The study received full ethical approval from the Department of Language and Communication Science Proportionate Review Committee at City St George's, University of London (ETH2425‐0828). It also received full ethics approval from the Ethics Committee of a specialist school and college that this study recruited participants from.

### Design

A cross‐sectional, between‐group study was adopted to examine and compare factors influencing wellbeing in three groups of young adults: those with post‐stroke aphasia, those with DLD and young adults with no history of language impairments. Data on wellbeing, language, cognition, health, emotional distress, social functioning (including social network, social connectedness, social support and community integration), and psychological resources (including self‐efficacy and self‐esteem) was collected. The feasibility and acceptability of the study design and the assessment tools were confirmed through a pilot study before data collection of the present study (see Supplementary file).

### Participants

To participate in the study, participants had to be aged 18–40 years, live in England, be capable of providing informed consent and have English as their first language. Individuals with co‐occurring diagnoses of autism, attention deficit hyperactivity disorder (ADHD) or neurological impairments other than stroke were excluded.

A wide recruitment approach involved outreach to charities, organizations, schools, support groups and healthcare professionals. The study was also promoted via websites, social media, personal networks and word‐of‐mouth. While no incentives were offered, travel expenses of participants were reimbursed. Participants were included in the aphasia and DLD groups based on self‐reported diagnosis, clinical records and language screening. Many individuals with aphasia were identified through aphasia registries, while participants with DLD had their diagnosis confirmed by teachers at a specialist school they attended.

During recruitment, 86 individuals showed interest in the study: 25 with aphasia, 22 with DLD and 39 typical. A total of 78 participants (19 with aphasia, 20 with DLD, 39 typical) participated in the research. Table 1 summarizes reasons for not taking part. Table 2 presents participants’ characteristics.

**TABLE 1:** Recruitment of participants.

Partaking	Typical group	Aphasia group	DLD group
Took part (*n*)	39	19	20
Expressed interest but did not take part	n.a.	6	2
Reasons for not taking part		Age (*n* = 1); English not first language (*n =* 1); cause of aphasia was BI (*n* = 1); living in USA (*n* = 1); was referred by another participant, but did not reply when contacted (*n* = 1); death (*n* = 1)	Initially said yes but at the end did not respond or could not make it (*n* = 2)

Abbreviations: BI, brain injury; DLD, developmental language disorders; *n*, number of participants; n.a., not applicable.

**TABLE 2:** Participant characteristics per group.

	Typical group	Aphasia group	DLD group
Characteristic	(*n* = 39)	(*n* = 19)	(*n* = 20)
**Age (years)**			
Mean (SD)	29.5 (5.63)	34.78 (5.05)	28.20 (7.17)
Range	18.03–40.06	25.11–40.08	18.02–40.11
**Gender, *n* (%)**			
Male	15 (38.5%)	9 (47.4%)	7 (37%)
Female	24 (61.5%)	10 (52.6%)	13 (65%)
**Aphasia severity, *n* (%)**			
Mild	n.a.	6 (31.6%)	n.a.
Moderate		10 (52.6%)	
Severe		2 (10.5%)	
Very severe		1 (5.3%)	
**Aphasia type, *n* (%)**			
Broca's	n.a.	7 (36.8%)	n.a.
Transcortical motor		3 (15.8%)	
Broca's/transcortical motor		1 (5.3%)	
Conduction		4 (21.1%)	
Anomic		4 (21.1%)	

*Note*: DLD, developmental language disorders; *n*, number of participants; n.a., not applicable; SD, standard deviation.

People with aphasia ranged in age from 25.11 to 40.08 years, with a mean of 34.78 (SD = 5.05) years; nine were males (47%) and 10 females (53%). In terms of aphasia there was a spread of types and severities, with the majority having moderate aphasia (*n* = 10; 52.6%) and over a third Broca's aphasia (*n* = 7; 36.8%).

The age of participants with DLD ranged from 18.02 to 40.11 years, with a mean of 28.20 years (SD = 7.17); 35% were male (*n* = 7) and 65% were female (*n* = 13).

Typical young adults ranged in age from 18.03 to 40.06 years with a mean of 29.5 (SD = 5.63) years. There were 15 males (38%) and 24 female (62%) participants in that group.

There was a significant difference in the age of the three groups *F*(2, 75) = 6.98; *p* = 0.002: those with aphasia were significantly older compared with participants with DLD (*p* = 0.004) and typical participants (*p* = 0.009).

### Procedure

Recruitment took place from 17 April 2018 to 13 September 2019. Interested individuals received project details and attended a meeting to give written consent after receiving clarifications. Eligibility was determined through language screening and case histories. The assessment of people with aphasia took 4 h on average, while the DLD group assessments lasted 3.5 h, usually spanning one to two sessions with an average interval of 13 days. All typical participants completed assessments in one session. Two participants, one with aphasia and one with DLD, had a significant other present on request. All sessions were recorded for scoring purposes.

### Measures

For eligibility screening, the Sentence Repetition and Verbal Fluency A (Animals) tasks from the Wechsler Individual Achievement Test—Second UK Edition (WIAT‐II^UK^; Wechsler, 2005) were used. These tasks, interpreted using adult norms, can distinguish typical language users from those with specific disorders, making them key indicators of language impairment (Rujas et al., 2021).

A total of 12 measures, chosen based on a systematic review, were used to evaluate wellbeing and contributing factors in the target populations. All measures had been validated or used previously with at least one clinical group. Typical and DLD participants completed 12 measures, while those with aphasia completed 13. The primary outcome measure was the Warwick‐Edinburgh Mental Well‐being Scale (WEMWBS; Tennant et al., 2007). Language, cognition, health, emotional distress, as well as social functioning and psychological resources measures were studied as potential factors influencing wellbeing. Additionally, aphasia severity and type were evaluated for the aphasia group using the Bedside Western Aphasia Battery—Revised (Bedside WAB‐R; Kertesz, 2007). This measure was included for descriptive purposes only and does not factor into the statistical comparisons between groups. Table 3 shows the measures used for each construct and their scoring system.

**TABLE 3:** Constructs evaluated, measures and score interpretation.

Category	Construct	Measure	Score interpretation
Language	Language screening	Wechsler Individual Achievement Test—Second UK Edition (WIAT‐II^UK^; Wechsler, 2005): Sentence Repetition and Word Fluency tasks of Oral Expression	Higher scoring shows better language skills
	Language testing	Wechsler Individual Achievement Test—Second UK Edition (WIAT‐II^UK^; Wechsler, 2005): Listening Comprehension and Oral Expression of Oral Language	Higher scoring shows better language skills
	Cognition	Cognitive Linguistic Quick Test (CLQT; Helm‐Estabrooks, 2001)	Higher scoring shows better cognitive skills
	Wellbeing	Warwick‐Edinburgh Mental Well‐being Scale (WEMWBS; Tennant et al., 2007)	Higher scoring shows higher levels of wellbeing
	General health	European Quality of Life—Five Dimensions (EQ‐5D‐5L; EuroQoL group, 2009, as cited in Herdman et al., 2011)	Higher scoring shows better health
Emotional health	Emotional distress	General Health Questionnaire – 28 Item (GHQ‐28; Goldberg & Hillier, 1979)	Higher scoring shows higher levels of emotional distress
Social functioning	Social network	Stroke Social Network Scale (SSNS; Northcott & Hilari, 2011)	Higher scoring shows better functioning in terms of social networks
	Subjective sense of Connectedness to the social world	Social Connectedness Scale—Revised (SCS‐R; Lee et al., 2001)	Higher scoring shows higher social connectedness
	Social support	Medical Outcome Studies Social Support Survey (MOS SSS; Sherbourne & Stewart, 1991)	Higher scoring shows better levels of social support
	Level of integration into the home and community	Community Integration Questionnaire (CIQ; Willer et al., 1993)	Higher scoring shows better social integration
Psychological resources	Self‐efficacy	General Self‐Efficacy Scale (GSE; Schwarzer & Jerusalem, 1995)	Higher scoring shows higher levels of self‐efficacy
	Self‐esteem	Self‐esteem—Rosenberg Self‐Esteem Scale (RSE; Rosenberg, 1965)	Higher scoring shows higher levels of self‐esteem
	Aphasia type & severity	Bedside Western Aphasia Battery—Revised (Bedside WAB‐R; Kertesz, 2007)	

To ensure communicative accessibility for participants with aphasia and DLD, all measures, aside from standardized language and cognition assessments, were adjusted, with larger fonts, one item per page and bolded key terms, adhering to best practice guidelines (Herbert et al., 2019). Participants’ responses were collected through interview‐based methods, with items read out, and participants having the option to indicate their responses by pointing if needed. The typical group completed scales independently, with the researcher assisting when needed.

### Data analysis

Data analysis was conducted using SPSS 25 for MAC OS. Participants’ characteristics and performance on each measure were summarized using descriptive statistics.

To compare groups on language, cognition and wellbeing, one‐way unrelated analyses of variance (ANOVAs) with post‐hoc comparisons and their non‐parametric counterpart, Kruskal‐Wallis tests, followed by Mann‐Whitney pairwise comparisons, were used. A Bonferroni adjustment was applied to control for Type I errors during post hoc tests, setting the new alpha (*α*) at 0.0167 (Field, 2018).

Effect sizes were determined in the comparative analyses. For ANOVAs, partial eta squared (*η*p^2^) interpretations were: 0.01 as small, 0.06 as medium and 0.14 as large. For the Mann‐Whitney tests, effect sizes were calculated as 𝑟 = 𝑧√𝑁 (Rosenthal, 1991, as cited in Field, 2018) and gauged using Cohen's criteria (Cohen, 1988):

- *r* = 0.10 (small effect): Explains 1% of total variance.
- *r* = 0.30 (medium effect): Explains 9% of the total variance.
- *r* = 0.50 (large effect): Explains 25% of the variance.

Correlation analyses were employed to identify potential factors influencing wellbeing across the three groups. Correlation strengths were based on Cohen's criteria (Cohen et al., 1998):

- *r* ≥ 0.50 as large.
- *r* = 0.30–0.49 as moderate.
- *r* = 0.10–0.29 as small.
- *r* < 0.10 as negligible.

## RESULTS

Descriptive information on missing data and factors explored as potential predictors of wellbeing is reported first, before addressing the research questions.

Small amounts of individual item data were missing from nine assessments. Overall, 0.90% of values were missing (*n* = 134) and 99.10% were completed (*n* = 14.764). A total of 77 of the 78 participants attended 100% of their arranged sessions. One person with aphasia did not complete the final session (i.e., four scales).

Table 4 presents descriptive information for each group on measures of health, emotional distress, social functioning and psychological resources scales. These were examined as potential factors influencing wellbeing, in addition to language and cognition which are presented in the following section.

**TABLE 4:** Descriptive statistics for health, emotional distress, social functioning scales and psychological resources scales.

	Typical group	Aphasia group	DLD group
Construct (scale)	(*n* = 39)	(*n* = 19)	(*n* = 20)
**General Health (EQ‐5D‐5L VAS)**			
Mean (SD)	80.18 (10.80)	72.68 (19.45)	85.15 (14.69)
Range (minimum–maximum)	54.00 (45.00–99.00)	70.00 (30.00–100.00)	50.00 (50.00–100.00)
Skewness	−0.77	−0.81	−1.38
**Emotional Distress (GHQ‐28)**			
Mean (SD)	18.31 (8.94)	24.89 (12.40)	20.40 (11.38)
Range (minimum–maximum)	42.00 (7.00–49.00)	42.00 (12.00–54.00)	42.00 (7.00–49.00)
Median (IQR)	16.00 (12.00–23.00)	20.00 (14.00–35.00)	16.00 (11.25–27.00)
Skewness	**1.64**	0.97	0.97
			
**Social network (SSNS)**			
Mean (SD)	58.57 (9.91)	55.86 (10.61)	52.42 (11.39)
Range (minimum–maximum)	39.84 (40.21–80.05)	35.26 (35.74–71.00)	54.84 (41.99–58.93)
Skewness	0.18	−0.36	−0.34
**Social connectedness (SCS)**	*n* = 38	*n* = 18	
Mean (SD)	97.61 (14.46)	80.06 (12.05)	81.40 (14.38)
Range (minimum–maximum)	66.00 (51.00–117.00)	44.00 (54.00–98.00)	60.00 (54.00–114.00)
Skewness	−1.32	−0.26	0.37
**Social support (MOS‐SSS)**		*n* = 18	
Mean (SD)	4.43 (0.62)	3.99 (0.68)	4.05 (0.78)
Range (minimum–maximum)	2.00 (3.00–5.00)	3.00 (2.00–5.00)	3.00 (2.00–5.00)
Skewness	−1.38	−0.59	−0.75
**Community integration (CIQ)**			
Mean (SD)	21.70 (3.22)	17.74 (3.25)	17.91 (4.70)
Range (minimum–maximum)	14.00 (15.00–28.00)	17.00 (3.00–20.00)	16.00 (11.00–26.00)
Skewness	−0.53	−0.59	0.17
**Self‐efficacy (GSE)**		*n* = 18	
Mean (SD)	32.34 (4.13)	26.83 (4.06)	26.60 (5.40)
Range (minimum–maximum)	18.00 (22.00–40.00)	16.00 (20.00–36.00)	23.00 (17.00–40.00)
Skewness	−0.30	−0.09	0.48
**Self‐esteem (RSE)**	*n* = 38		
Mean (SD)	32.08 (5.23)	28.78 (4.18)	29.15 (5.21)
Range (minimum–maximum)	23.00 (17.00–40.00)	15.00 (20.00–35.00)	19.00 (21.00–40.00)
Skewness	−0.55	−0.41	0.23

*Note*: Skewness values in bold indicate skewed data (values outside ±1.5 range).

DLD, developmental language disorders; minimum–maximum, minimum–maximum; *n*, number of participants; SD, standard deviation.

### How do the language and cognitive skills of young adults with aphasia, DLD and typical individuals compare?

#### Background language assessments

Descriptive statistics for Language as assessed with the WIAT‐II^UK^ are presented separately for each group in Table 5.

**TABLE 5:** Descriptive statistics of WIAT‐II^UK^ scores per group.

	Typical group	Aphasia group	DLD group
WIAT‐II^UK^ domain	(*n* = 39)	(*n* = 19)	(*n* = 20)
**Listening comprehension**			
Mean (SD)	97.95 (14.43)	65.95 (25.97)	73.90 (19.90)
Range (minimum–maximum)	58.00 (56.00–114.00)	66.00 (40.00–106.00)	62.00 (40.00–102.00)
Skewness	−1.41	0.64	−0.20
**Oral expression**			
Mean (SD)	112.13 (11.88)	77.11 (16.14)	90.55 (15.89)
Range (minimum–maximum)	51.00 (84.00–135.00)	60.00 (41.00–101.00)	53.00 (62.00–115.00)
Skewness	−0.36	−1.09	−0.37
**Oral language composite**			
Mean (SD)	105.72 (13.13)	71.16 (18.22)	81.60 (16.17)
Range (minimum–maximum)	52.00 (75.00–127.00)	61.00 (41.00–102.00)	58.00 (51.00–109.00)
Skewness	−0.42	0.05	−0.23

*Note*: DLD, developmental language disorders; *n*, number of participants; SD, standard deviation; WIAT‐II^UK^: Wechsler Individual Achievement Test—Second UK Edition.

There was a significant difference between the three groups on Listening Comprehension [Welch Adjusted *F*(2, 32.82) = 20.21, *p* < 0.001, *η*p^2^ = 0.37], Oral Expression [*F*(2, 75) = 43.40, *p* < 0.001, *η*p^2^ = 0.54] and Oral Language Composite [*F*(2, 75) = 37.94, *p* < 0.001, *η*p^2^ = 0.50]. In all these measures, the typical group had the highest scores and people with aphasia the lowest scores. In post‐hoc comparisons (Scheffe), typical adults scored significantly better than those with DLD (*p* < 0.001) and those with aphasia (*p* < 0.001), but the only significant difference between those with DLD and those with aphasia was on Oral Expression (*p* = 0.015), with the aphasia group scoring less favourably.

#### Background cognition assessments

Descriptive statistics for Language, Non‐linguistic Cognition and Composite Cognitive Severity scores as assessed with the Cognitive Linguistic Quick Test (CLQT; Helm‐Estabrooks, 2001) are presented in Table 6.

**TABLE 6:** Descriptive statistics of cognitive scores per group.

CLQT domain	Typical group	Aphasia group	DLD group
Possible score range	(*n* = 39)	(*n* = 19)	(*n* = 20)
**Language, 0–37**			
Mean (SD)	35.36 (1.50)	25.08 (6.25)	31 (3.31)
Range (minimum–maximum)	5.00 (32.00–37.00)	24.00 (8.00–32.00)	12.00 (24.00–36.00)
Skewness	−0.086	−1.26	−0.45
**Non‐linguistic cognition, 0–49**			
Mean (SD)	43.87 (3.41)	40.97 (8.77)	37.6 (6.46)
Range (minimum–maximum)	17.00 (32.00–49.00)	32.00 (17.00–49.00)	23.00 (24.00–47.00)
Median (IQR)	45.00 (42.00–46.00)	44.00 (38.5–47)	38.50 (32.25–43.0)
Skewness	−1.18	**−1.83**	−0.39
**Composite severity, 1–4**			
Mean (SD)	3.99 (0.45)	3.40 (0.86)	3.56 (0.54)
Range (minimum–maximum)	0.20 (3.80–4.00)	2.80 (1.20–4.00)	1.80 (2.20–4.00)
Median (IQR)	4.00 (4.00–4.00)	3.80 (2.80–4.00)	3.80 (3.25–4.00)
Skewness	**−4.23**	−1.47	−1.09

*Note*: Skewness values in bold colour indicate skewed data (values outside ±1.5 range).

CLQT, Cognitive Linguistic Quick Test; DLD, developmental language disorder; *n*, number of participants; SD, standard deviation; IQR, interquartile range.

There was a significant difference between the three groups on CLQT Language scores (Welch Adjusted *F*(2, 27.62) = 51.33, *p* < 0.001, *η*p^2^ = 0.58). Language scores differed significantly among all three groups (*p* < 0.001 in all comparisons), with the typical group showing the highest and the aphasia group the lowest scores. In terms of CLQT non‐linguistic and Composite Cognitive Severity, there was a significant difference between the groups (*H*(2) = 12.80, *p* = 0.002; *H*(2) = 21.75, *p* < 0.001 respectively).

In post‐hoc comparisons, typical adults scored significantly better than those with DLD in both CLQT Non‐linguistic (*U* = 162.00, *p* < 0.001; *r* = 0.77) and in Composite Cognitive Severity (*U* = 205.00, *p* < 0.001; *r* = 0.55), while the two clinical groups showed no differences in these domains, that is, *U* = 116.00, *p* = 0.037; *r* = 0.33 (based on the Bonferroni adjusted *α*) and *U* = 184.50, *p* = 0.869; *r* = 0.03 respectively. People with aphasia showed significantly worse Composite Cognitive Severity compared with typical adults (*U* = 185.50, *p* < 0.001; *r* = 0.57), but no significant difference was found in the Non‐Linguistic domain (*U* = 324.00, *p* = 0.439; *r* = 0.10).

### How does the wellbeing of young adults with aphasia, DLD and typical individuals compare?

Descriptive statistics for wellbeing as assessed with WEMWBS are presented in Table 7. The difference between the three groups in WEMWBS scores was not significant, that is, *F*(2, 75) = 1.47, *p* = 0.236. The effect size was small (*η*p^2^ = 0.04).

**TABLE 7:** Descriptive Statistics for wellbeing per group.

WEMWBS	Typical group	Aphasia group	DLD group
Possible score range (14–70)	(*n* = 39)	(*n* = 19)	(*n* = 20)
Mean (SD)	52.36 (7.41)	49.00 (7.19)	50.10 (7.82)
Range (minimum–maximum)	35.00 (31.00–66.00)	25.00 (39.00–64.00)	26.00 (39.00–65.00)
Skewness	−0.77	0.36	0.38

*Note*: DLD, developmental language disorder; *n*, number of participants; SD, standard deviation; WEMWBS, Warwick‐Edinburgh Mental Wellbeing Scale.

### What are the shared and different influencing factors of wellbeing between young adults with aphasia, DLD and typical individuals?

Demographic variables were not correlated to wellbeing. Age was not linked with wellbeing in any group (typical: *r*(39) = −0.20, *p* = 0.903; aphasia: *r*(19) = −0.174, *p* = 0.48; DLD: *r*(20) = −0.004, *p* = 0.987). Men and women's wellbeing scores did not differ [*t*(76) = −0.82, *p* = 0.41]. Living Arrangements, whether living alone or with others, did not affect wellbeing [*t*(72) = −1.41, *p* = 0.16]. Wellbeing was also not significantly different across Marital Status (*F*(2, 73) = 2.39, *p* = 0.09). Thus, these factors were not analysed further. Table 8 shows correlations between wellbeing and the Language and Cognition scores, separately for each group.

**TABLE 8:** Correlation results between wellbeing and language and cognition variables.

	Listening comprehension	Oral expression	Oral language	Language	Non‐linguistic	Composite cognition
Group	WIAT‐II	WIAT‐II	WIAT‐II	CLQT	CLQT	CLQT
Typical	*r* = −0.197	*r* = −0.035	*r* = −0.129	*r* = −0.138	*r* = −0.086	*ρ* = −0.036
*p*‐value	0.229	0.834	0.436	0.403	0.605	0.827
*N*	39	39	39	39	39	39
Aphasia	*r* = −0.030	*r* = 0.104	*r* = 0.030	*r* = −0.082	*ρ* = 0.190	*r* = −0.099
*p*‐value	0.902	0.672	0.903	0.740	0.435	0.688
*N*	19	19	19	19	19	19
DLD	*r* = −0.400	*r* = **−0.460**	*r* = **−0.450**	*r* = ***−0.572***	*r* = −0.187	*r* = ***−0.469***
*p*‐value	0.081	0.041	0.046	0.008	0.431	0.037
*N*	20	20	20	20	20	20

*Note*: Significant correlations at the level of 0.05 (two‐tailed) are shown in bold. Italics indicate a strong and underlined a medium relationship.

CLQT, Cognitive Linguistic Quick Test; DLD, developmental language disorder; *N*, sample size; *r*, Pearson correlation coefficient; WIAT‐II, Wechsler Individual Achievement Test—Second Edition; *ρ*, Spearman correlation coefficient.

#### Language and cognitive characteristics and wellbeing

Looking at the relationship of wellbeing with Language (WIAT‐II^UK^) and Cognition (CLQT) measures, no significant relationships were found in the typical and aphasia groups. All relationships were unimportant or small.

In the DLD group, wellbeing was significantly negatively associated with the Language domain of CLQT [*r*(20) = −0.57, *p* = 0.008]. Negative, medium and significant relationships were also found between wellbeing and: Overall Cognition as measured with the CLQT [*r*(20) = −0.47, *p* = 0.037], WIAT‐II^UK^ Oral Expression [*r*(20) = −0.46, *p* = 0.041], and WIAT‐II^UK^ Oral Language Total [*r*(20) = −0.45, *p* = 0.046] for the DLD group, indicating that the better participants’ cognitive and language skills were, the lower they rated their wellbeing.

#### Health, emotional, and psychosocial variables and wellbeing

Correlation results between wellbeing and General Health, Emotional Distress, social functioning variables and psychological resources variables are presented in Table 9.

**TABLE 9:** Correlation results between wellbeing and health, emotional, social and personal variables.

Group	General health	Emotional distress	Social network	Social connectedness	Social support	Community integration	Self‐efficacy	Self‐esteem
Typical	*r* = ***0.521***	*ρ* = **−0.346**	*r* = 0.185	*r* = ***0.696***	*r* = **0.350**	*r* = 0.064	*r* = **0.422**	*r* = ***0.727***
*p*‐value	.001	0.031	0.259	<0.001	0.029	0.699	0.008	<0.001
*N*	39	39	39	38	39	39	38	38
Aphasia	*r* = **0.476**	*r* = ***−0.547***	*r* = 0.326	*r* = 0.324	*r* = **0.493**	*r* = 0.193	*r* = 0.280	*r* = 0.382
*p*‐value	.040	0.027	0.173	0.189	0.038	0.429	0.261	0.117
*N*	19	19	19	18	18	19	18	18
DLD	*r* = ***0.570***	*r* = ***−0.538***	*r* = 0.108	*r* = 0.440	*r* = ***0.568***	*r* = −0.002	*r* = −0.258	*r* = ***0.566***
*p*‐value	.009	0.014	0.650	0.052	0.009	0.995	0.272	0.009
*N*	20	20	20	20	20	20	20	20

*Note*: Significant correlations at the level of 0.05 (two‐tailed) are shown in bold. Italics indicate a strong and underlined a medium relationship.

*Note*: DLD, developmental language disorder; *N*, sample size; *r*, Pearson correlation coefficient; *ρ*, Spearman correlation coefficient.

*Aphasia group*. In the aphasia group, there was a significant, strong, negative relationship between wellbeing and Emotional Distress [*r*(19) = −0.55; *p* = 0.015]. Conversely, people who had more Social Support and better General Health also reported better wellbeing, with a significant, positive, medium relationship that is, *r*(18) = 0.49 (p = 0.038) and *r*(19) = 0.48 (*p* = 0.040) respectively.

*DLD group*. Similarly, in the DLD group, there was also a significant, strong, negative relationship between wellbeing and Emotional Distress [*r*(20) = −0.54, *p* = 0.014)]; and a significant, strong, positive relationship between wellbeing and General Health [*r*(20) = 0.57, *p* = 0.009], Social Support [*r*(20) = 0.57, *p* = 0.009], and Self‐esteem [*r*(20) = 0.57, *p* = 0.009)].

*Typical group*. In the typical group, wellbeing was significantly and strongly positively correlated with Self‐esteem [*r*(38) = 0.73; *p* < 0.001], Social Connectedness [*r*(38) = 0.70; *p* < 0.001], and General Health [*r*(39) = 0.52; *p* < 0.001]. A significant medium and positive correlation was shown between scores of wellbeing and Self‐efficacy [*r*(38) = 0.42; *p* = 0.008] and wellbeing and Social support [*r*(39) = 0.35; *p* = 0.029], showing that participants who felt more self‐efficient and had more support, reported better wellbeing.

In summary, comparing the two clinical groups, General Health, Emotional Distress and Social Support were common influencing factors of wellbeing. However, in the DLD group, wellbeing was also correlated with Self‐esteem, Language and Cognition domains. For both groups, the relationship of wellbeing with Social Network, Social Connectedness, Social Integration and Self‐efficacy was not significant.

## DISCUSSION

This exploratory study is the first to directly compare language, cognition and wellbeing across young adults with aphasia and those with DLD and factors influencing wellbeing.

### Language and cognition performance

Clinical groups scored lower than typical peers in language and cognition, including subdomains. Lower scores in expressive, receptive, and overall language were expected due to definitions and eligibility criteria for aphasia and DLD as language disorders. Differences between the DLD group and typical peers in language scores indicate continued linguistic challenges into young adulthood, supporting evidence that DLD persists beyond adolescence (Botting, 2020; Clegg et al., 2005).

The only significant differences between the clinical groups were in expressive language (WIAT‐IIUK) and CLQT language scores, with the aphasia group scoring lower. While our analyses of broad language and cognitive categories aimed to understand potential variations in wellbeing, these specific language findings highlight the need for more focused investigation.

Individuals with DLD and those with aphasia can exhibit different patterns and levels of language abilities, and the nature and extent of these difficulties can vary widely between individuals (Botting, 2020; Conti‐Ramsden et al., 2016, 2018). While the language scores in our study reflect a range of abilities, this variability is particularly relevant when considering how language challenges impact psychosocial wellbeing. Difficulties in expressive, receptive or discourse‐level abilities can significantly influence social participation, emotional health and overall wellbeing within these groups (Botting, Durkin et al., 2016; Hilari & Northcott, 2006). Future research could further explore the relationship between specific linguistic domains and psychosocial outcomes to identify patterns of strengths and weaknesses that may contribute to wellbeing differences or similarities across these populations. This approach would provide a better understanding of how language abilities relate to broader life experiences.

The present findings also suggest a need for further exploration of domain‐specific versus domain‐general impairments. Specifically, our results indicate that cognitive impairment in aphasia may primarily be linguistic in nature with deficits stemming from difficulties in accessing and processing language. In contrast, in DLD, a more generalized cognitive impairment might be present, potentially involving broader deficits in attention, memory and executive functioning. These findings are in accordance with previous literature (Bishop et al., 2014; Botting, 2005). However, this distinction requires further investigation to better understand the underlying mechanisms of impairment in each condition. Exploring these differences could help clarify whether cognitive impairments in these groups arise independently or as a consequence of their language difficulties.

### Wellbeing status and factors influencing wellbeing

Wellbeing ratings of young adults with aphasia and DLD were similar to those of their typical peers. Our results align with DLD studies in the UK (Conti‐Ramsden et al., 2016), the United States (Records et al., 1992) and Canada (Johnson et al., 2010). However, our study, to the best of our knowledge is the first to investigate and indicate similar wellbeing between young adults with aphasia and their typical peers. More research is needed in this area to add confidence to our findings.

Our findings indicate that wellbeing is a complex and internal state, influenced by several factors and may not directly reflect objective circumstances (Diener, 1984) such as language disorders and associated socio‐emotional difficulties. We propose that individuals with language disorders like aphasia and DLD can experience high levels of wellbeing, just as neurotypical individuals may have low wellbeing without apparent disorders.

Young adult DLD research aligns with this perspective. Arkkila et al. (2008) found no overall QoL difference between DLD and typical groups but noted challenges in QoL dimensions like speech, activities, distress and mental function. Similarly, other studies suggest that despite life difficulties, overall QoL may not differ significantly in DLD individuals (Johnson et al., 2010; Records et al., 1992). These findings align with Donovan et al. (2002), who view disability as an intriguing paradox; ‘against all odds’, disabled individuals appear to adapt to the adverse circumstances of their impairment, demonstrating a certain level of happiness.

#### Shared influencing factors

In our sample, Social Support, Health Status and Emotional Distress were shared influencing factors of wellbeing across all groups, underlining their universal role in shaping wellbeing.

Looking at those in more detail, there is no research on the relationship between social support and wellbeing in young adults with aphasia. However, studies with older individuals with post‐stroke aphasia indicate a strong link between social support and QoL (Hilari et al., 2012), which is expected, as sudden aphasia often increases reliance on caregivers and community networks (Hilari & Northcott, 2006). Young adults with DLD receive more assistance than typical peers (Botting, Durkin et al., 2016). Supportive social environments significantly affect their wellbeing, particularly during challenging times (Conti‐Ramsden et al., 2016). Previous research found that strong social support at age 19 correlated with higher QoL at age 25, suggesting family support as a protective factor (Johnson et al., 2010). Positive wellbeing ratings in our clinical groups may result from supportive environments, but further analysis is needed to explore this hypothesis since our study did not assess the level of support in our groups.

General health was crucial for wellbeing in our aphasia group, possibly due to the important stroke‐related health issues. This connection is less studied in younger individuals. Hinckley (1998) found no significant health‐life satisfaction link in middle‐aged individuals with aphasia. However, participants of that study perceived themselves as heathy, which may affect results. Also, Hinckley's single‐question life satisfaction measure raises validity concerns. Conversely, research on young adults with DLD, and similar methodology to ours, aligns with our findings, emphasizing self‐reported health as the most significant wellbeing predictor, explaining 21% of wellbeing variance, regardless of employment or relationship status (Conti‐Ramsden et al., 2016).

Emotional health significantly impacted wellbeing across all groups, especially the aphasia group, displaying a strong correlation. This is unsurprising, as according to clinicians and service users the loss of skills in those with acquired language impairment can lead to feelings of sadness and worry (Conti‐Ramsden & Botting, 2008). Emotional distress in older aphasia patients has been linked to lower QoL (Hilari et al., 2012). However, a study comparing predictors of QoL in older and younger stroke patients, including those with aphasia, found that while depression was closely related to QoL in older‐onset patients, it did not predict overall QoL or its subdomains in younger stroke survivors (Kim et al., 2005). However, they focused on stroke, not exclusively on aphasia, and post‐stroke depression was very infrequent in that group.

Young adults with DLD consistently report higher depression and anxiety than controls (e.g., Arkkila et al., 2008; Botting, Durkin et al., 2016; Clegg et al., 2005). Longitudinal studies on children with DLD reveal varying patterns of mental health issues in young adulthood, evolving with life stages (Conti‐Ramsden et al., 2018). Interestingly, some report curvilinear patterns, initially decreasing, then increasing symptoms (Botting, Durkin et al., 2016). Despite extensive emotional health research in young adults with DLD, our study is the first that has demonstrated its impact on overall wellbeing.

#### Differentiating influencing factors

Noteworthy is the contrasting relationship between wellbeing and language in our clinical groups, despite no significant differences in profiles of language, cognition, wellbeing outcomes and correlates. Strong language‐wellbeing links were found in the DLD group, who grew up with language challenges. In contrast, despite lower oral expression linguistic performance, language performance had limited effects on wellbeing in the aphasia group.

In related research is aphasia, in multiple regression analyses, aphasia did not predict overall QoL; instead, predictors included degree of disability and dependence, motor dysfunction, dysarthria and economic status (Kim et al., 2005). This supports our suggestion that aphasia's negative impact on overall QoL may be mitigated by other factors when studied together, like emotional distress and general health in our study.

Conti‐Ramsden and Botting (2008) discuss that a lack of clear association between early‐life language scores and mental health in young adults with DLD challenges interpretations of poor language's direct developmental causality for emotional health difficulties. They support that ongoing poor communicative experiences do not lead to increased depression or anxiety; instead, the association appears linked to the disorder itself. Interestingly, this challenges the notion of a direct developmental causal relationship between early language difficulties and later emotional health outcomes. Instead, it is suggested that atypical development occurring at very early stages of life may give rise to multiple deficits that remain latent and only become apparent as individuals face increasing social, emotional and communicative demands later in life (Conti‐Ramsden & Botting, 2008). That different focus may also explain differences between our and Kim et al. (2005) findings, where we examined language performance and wellbeing while they examined the disorder itself and QoL.

Differences also exist in the link between self‐esteem and wellbeing in the clinical groups. The aphasia group lacks a significant connection between these factors, consistent with research showing no significant link between self‐esteem and language functioning in stroke patients with language impairments (Vickery, 2006) and no evidence of low self‐esteem shortly after stroke onset (Bakheit et al., 2004). In contrast, a significant correlation emerged between these constructs in the DLD group. Durkin et al. (2017) reported lower self‐esteem, increased shyness and reduced social self‐efficacy in individuals with DLD as well as an association between higher language scores at age 17 and increased self‐esteem, social self‐efficacy and decreased shyness at age 24.

Language is crucial for learning and socialization in childhood and adolescence, while self‐esteem primarily develops during these stages, influenced by family environment (Harter, 2015), academic performance (Zheng et al., 2020), and peer interactions. Persistent language challenges in individuals with DLD are expected to impact self‐perception, affecting feelings and social functioning in adulthood (Durkin et al., 2017). In contrast, in aphasia, which emerges suddenly in adulthood, self‐esteem may not be directly linked to wellbeing, possibly because self‐esteem is more stable at this life stage (Trzesniewski et al., 2003), rooted in past linguistic competence and roles.

In summary, our findings underscore early life experiences and developmental factors in shaping self‐esteem and wellbeing in individuals with DLDs. They also highlight resilience and adaptive strategies’ potential to mitigate the impact, particularly when disorders are acquired later in life.

### Limitations and future research

The current study has limitations that could be considered in future research. While the data collection was thorough, a larger and more diverse sample in the clinical groups would have allowed for a more comprehensive exploration of relationships between variables, enabling us to predict outcomes and quantify the strength of these relationships. Notably, the DLD sample was primarily recruited from a single school, which may limit the generalizability of our findings. Future studies should aim to include participants from a broader range of schools and settings to enhance the diversity and representativeness of the sample. A larger sample size could also enable more complex analyses, like moderation and mediation, to better understand wellbeing predictors. This would be particularly useful in better understanding the relationship between language abilities and wellbeing in the aphasia group. Botting, Durkin et al. (2016) showed that language and mental health's relation is not straightforward, with self‐efficacy mediating it, indicating complexities that merit further study. While we accounted for several factors, we did not compare health and support levels between groups and thus we cannot determine whether these levels are lower than those of typically developing participants. Looking at such differences can contribute to observed patterns and aid in the interpretation of findings. In addition, we examined and excluded individuals with DLD who also presented with autism and/or ADHD. We prioritized these conditions in the DLD group due to their high co‐occurrence rates with DLD and significant impact on language development. Other co‐occurring conditions, including neurodevelopmental and mental health issues, should also be considered in future research.

### Clinical implications

In terms of clinical implications, understanding the factors that influence wellbeing is essential for tailoring effective services and policies (ONS, 2015). Young adults having aphasia and DLD often lack sufficient support due to limited evidence. Based on our findings, it is essential for speech and language therapists (SLTs) to continue adopting a holistic care approach, which is fundamental to their role. This approach should thoroughly consider psychological and environmental factors in the assessment and therapy process to ensure comprehensive care.

For individuals with aphasia, structured psychological care is crucial (Kneebone, 2016), along with promoting social connections (Northcott et al., 2016). Addressing emotional wellbeing in aphasia requires a multidisciplinary approach. Change can be initiated through specialized training for healthcare professionals in communication support, mood assessment and treatment, adapting physical environments and providing accessible resources (Baker et al., 2021). For individuals with DLD, therapists should adopt a comprehensive approach, targeting language and associated limitations across various life domains. Collaboration with psychologists and educators is essential. Interventions can benefit from applying communication skills in functional contexts relevant to young adults, such as workplace interactions, social settings and community involvement, in addition to peer interactions in educational settings.

The Intercollegiate Stroke Working Party (2023) emphasizes the unique unmet needs of young stroke patients. It highlights the importance of recognizing and managing their specific physical, psychological and social needs. These needs include vocational rehabilitation and childcare. Similarly, teenagers and young adults with DLD express concerns about the lack of age‐appropriate youth clubs (Myers et al., 2011). Local service providers must tailor offerings for age‐specific requirements, promote social interaction and support language development. Psychological services like counselling, peer support and vocational rehabilitation are particularly relevant for improving young adults’ outcomes, with collaboration between SLTs and affected individuals being vital for developing effective services.

## CONCLUSIONS

This study has offered insights into the intricate relationships between wellbeing and its determinants among young adults with aphasia and those with DLD. While both clinical groups exhibited positive wellbeing levels, the analysis has unveiled noteworthy distinctions in the connections between wellbeing and other influential factors, including language performance, which had an effect for those with DLD but not those with aphasia. The outcomes highlight how the nature of the disorder shapes wellbeing differently.

Our innovative methodology of simultaneously studying two language‐impaired populations with different aetiologies has yielded invaluable insights, laying the groundwork for more focused investigations. Theoretically, the study emphasizes the need for further research into wellbeing in the context of language disorders. Our approach underscores the importance of comprehensively analysing the interconnections between linguistic, cognitive and social‐psychological aspects related to wellbeing. It encourages a deeper exploration of the role of early‐life experiences in relation to wellbeing, while it underscores the significance of psychological resources and environmental factors in the development of wellbeing, as well as their potential role as protective factors against adverse outcomes in language disorders.

Young adults are moving out of the services that supported them as children and adolescents, whereas adult systems (e.g., the adult health care system, the employment sector and the justice system) may not be well suited to supporting their needs (National Research Council, 2013). Understanding factors influencing wellbeing of people with aphasia and DLD in young adulthood can guide the development of customized support services and holistic therapies specific to their needs. For example, according to Intercollegiate Stroke Working Party (2023), younger adults often experience strokes due to uncommon causes, and their rehabilitation may need to focus specifically on work‐related issues and parenting responsibilities. Additionally, their social needs and expectations can differ from those of older stroke patients. Understanding and managing the distinct physical, psychological and social needs of younger patients such as vocational rehabilitation and childcare (Intercollegiate Stroke Working Party, 2023), during this critical life period is essential. This can help those young people navigate life's challenges more effectively, leading to fulfilling, meaningful lives.

## CONFLICT OF INTEREST STATEMENT

The authors report no conflicts of interest.

## Supporting information



Supporting Information

## Data Availability

The data that support the findings of this study are available from the corresponding author upon request. The data are not publicly available due to privacy or ethical restrictions.

## ENDNOTE

1 In this study, we refer to young adults as individuals between the ages of 18 and 40, distinguishing them from older adults, who are more commonly the focus in aphasia literature. Also, the term ‘young adult’ in stroke research does not align exactly with its use in DLD research. To address this, we have adopted a compromise age range of 18–40 years.
