# Supplementary material for: Comparing factors influencing wellbeing in young adults with aphasia and young adults with developmental language disorder
Source: Int J Lang Commun Disord. 2025 Mar 6;60(2):e70020. doi: 10.1111/1460-6984.70020 (PMC11885860; doi:10.1111/1460-6984.70020)
Supplement: Supplementary file 1 — Supporting Information [file JLCD-60-0-s001.docx]

**Pilot study:**

**Feasibility and acceptability of testing materials and procedures for the study**

**Background**

The present pilot study used data from undergraduate student projects to conduct a preliminary comparison of the wellbeing between young adults with aphasia and those with developmental language disorder (DLD). It also aimed to explore feasibility and acceptability of testing materials and procedures due to the absence of previous relevant literature.

**Methods**

A cross-sectional study employing a between-group approach was conducted. Participants, aged 18-35, included individuals with aphasia, DLD, and typically developing (TD) individuals. Eligibility criteria required English as their first language, residency in the UK, adequate comprehension levels for consent, and no current diagnoses of learning disabilities, autism, or neurological disorders. Ethical approval was obtained, and participants provided written consent. Individual assessments of language, cognition, and psychosocial aspects were conducted.

Assessment measures used included:

- Language Assessment: The Frenchay Aphasia Screening Test (FAST; Enderby, Wood and Wade, 1987) evaluated language skills (comprehension, expression, reading, writing).
- Cognition Assessment:
  - Raven's Coloured Progressive Matrices (RCPM; Raven, 1995) assessed non-verbal intelligence.
  - Delis-Kaplan Executive Function System Battery (DKEFS; Delis et al., 2001) evaluated executive function, including Trail Making, Colour Word Fluency (Stroop, 1935), and Verbal Fluency.
  - The Odd One Out task measured visuospatial working memory.
- Psychosocial Assessment: Various measures were used, including EQ-5D-5L (EuroQol Group, 2009; as cited in Herdman et al., 2011) for general health, Stroke Social Network Scale (SSNS; Northcott & Hilari, 2013), Medical Outcome Studies Social Support Survey (MOS-SSS; Sherbourne & Stewart, 1991), and Social Connectedness Scale (SCS; Lee & Robbins, 1995) for social aspects. Emotional health was assessed using the General Health Questionnaire (GHQ-28; Goldberg, 1979), and self-efficacy with the General Self-Efficacy Scale (GSE; Jerusalem & Schwarzer, 1995).

To test feasibility, student projects were reviewed for recruitment and duration to administer measures. Accessibility was evaluated by exploring scoring on measures, e.g., ceiling or floor effects, as well an exploration of missing data. Anecdotal participant comments on acceptability were also considered.

**Findings and** **Implications for main study**

Recruitment challenges were encountered for both clinical groups. The first student group aimed for 30 participants but only managed to recruit 11 with DLD from a specialised school and two with aphasia from a support group, while they recruited 15 TD participants with no challenges. The second group aimed for 45 participants but recruited 33, including 30 TD, two with DLD, and one with aphasia. To address these recruitment challenges, the main project used a broader recruitment strategy and extended the recruitment period over 1.5 years.

Participants generally handled the testing procedure well. In terms of the session format, during the pilot study, where assessments lasted up to two hours, some participants expressed a need for breaks, while others declined the offered breaks. This suggested that researchers should be flexible in session duration, breaks, and segmentation to accommodate varying needs. Indeed, most participants with DLD and aphasia in the main project completed assessments in two sessions as preferred.

Regarding the acceptability of the testing materials, missing data occurred. Seven individuals with DLD, one with aphasia, and one TD participant did not complete one or more standardised tasks. Five individuals with DLD declined the Trial Making switching task. Additionally, two participants from the TD group, one from the DLD group, and two from the aphasia group reached the ceiling in RCPM scores. Data from two participants with DLD was missing as they found the tasks difficult. In one set of student projects, the Verbal Fluency Task, Colour Word Interference (Condition 4), and the EQ-5D-5L were left incomplete, leading to missing data for 30 TD participants, two with DLD, and one participant with aphasia. However, except for one TD participant omitting the Somatic Symptoms category of GHQ-28 and another skipping the Social Dysfunction category, all other measures were completed by all participants. Some participants also felt compelled to choose an answer when unsure between two ratings for the scales. Furthermore, the prevalent ceiling effect among the majority of DLD participants in the FAST highlighted the necessity of employing a different language screening test in the main study.

These observations raised questions about the appropriateness of these cognitive and language measures for our target populations and thus in the main project, different language and cognition measures were used, specifically the Wechsler Individual Achievement Test (WIAT-II^UK^; Wechsler, 2005) and the Cognitive Linguistic Quick Test (CLQT; Helm-Estabrooks, 2001). All participants completed those measures successfully with no missing data.

Participants found that the SSNS has limited response options for questions about participant contact with their social network, like email, letter, or phone. Many used WhatsApp, texting, and social media for communication, not covered in the choices. To ensure accuracy, in the main project, we clarified that "phone contact" includes mobile apps as suggested by the scale developers. Participants also noted traditional letters are rare, using emails mainly for work or colleagues, which was also clarified during the main study.

In conclusion, the pilot study aimed to assess feasibility, measure participants' ability to navigate the study, and evaluate the acceptability of testing materials. Overall, findings were positive, with the need to address specific issues ensuring improved accessibility and practicability during the assessment process in the main study.

**References**

1. Delis, D. C., Kaplan, E., & Kramer, J. H. (2001). Delis-Kaplan Executive Function System: Technical Manual. San Antonio, TX: Harcourt Assessment Company.
2. Enderby, P. M., Wood, V., & Wade, D. (1987). Frenchay Aphasia Screening Test (FAST) Test Manual. Whurr Publishers.
3. Goldberg, D. P., & Hillier, V. F. (1979). A scaled version of the General Health Questionnaire. Psychological Medicine, 9(1), 139–145.
4. Helm-Estabrooks, N. (2002). Cognition and aphasia: A discussion and a study. Journal of Communication Disorders, 35(2), 171–186.
5. Herdman, M., Gudex, C., Lloyd, A., Janssen, M. F., Kind, P., Parkin, D., Bonsel, G., & Badia, X. (2011). Development and preliminary testing of the new five-level version of EQ-5D (EQ-5D-5L). Quality of Life Research, 20(10), 1727–1736.
6. Lee, R. M., & Robbins, S. B. (1995). Measuring belongingness: The Social Connectedness and the Social Assurance scales. Journal of Counseling Psychology, 42(2), 232–241.
7. Northcott, S., & Hilari, K. (2013). Stroke Social Network Scale: Development and psychometric evaluation of a new patient-reported measure. Clinical Rehabilitation, 27(9), 823–833.
8. Raven, J. C. (1995). Court, JH, & Raven, J. (1990). Coloured progressive matrices, 10-11.
9. Schwarzer, R., & Jerusalem, M. (1995). Generalized Self-Efficacy scale. In J. Weinman, S. Wright, & M. Johnston (Eds.), Measures in health psychology: A user’s portfolio. Causal and control beliefs (pp. 35-37). Windsor, England: NFER-NELSON.
10. Sherbourne, C. D., & Stewart, A. L. (1991). The MOS social support survey. Social Science & Medicine, 32(6), 705–714.
11. Stroop, J. R. (1935). Studies of interference in serial verbal reactions. Journal of Experimental Psychology, 18(6), 643–662.
12. Wechsler, D. (2005). Wechsler individual achievement test–Second UK edition. The Psychological Corporation.
